# Intranasal antisepsis to reduce influenza virus transmission in an animal model

**DOI:** 10.1111/irv.13035

**Published:** 2022-10-12

**Authors:** Nassima Gaaloul ben Hnia, Mathew Kipkemboi Komen, Katie F. Wlaschin, Ranjani V. Parthasarathy, Kevin D. Landgrebe, Nicole M. Bouvier

**Affiliations:** ^1^ Department of Microbiology Icahn School of Medicine at Mount Sinai New York New York USA; ^2^ Corporate Research Materials Laboratory 3M St. Paul Minnesota USA; ^3^ Medical Solutions Division 3M St. Paul Minnesota USA; ^4^ Division of Infectious Diseases, Department of Medicine Icahn School of Medicine at Mount Sinai New York New York USA

**Keywords:** guinea pig, influenza, iodine, transmission

## Abstract

**Background:**

Seasonal influenza annually causes significant morbidity and mortality, and unpredictable respiratory virus zoonoses, such as the current COVID‐19 pandemic, can threaten the health and lives of millions more. Molecular iodine (I_2_) is a broad‐spectrum, pathogen‐nonspecific antiseptic agent that has demonstrated antimicrobial activity against a wide range of bacteria, virus, and fungi.

**Methods:**

We investigated a commercially available antiseptic, a non‐irritating formulation of iodine (5% povidone‐iodine) with a film‐forming agent that extends the duration of the iodine's antimicrobial activity, for its ability to prevent influenza virus transmission between infected and susceptible animals in the guinea pig model of influenza virus transmission.

**Results:**

We observed that a once‐daily topical application of this long‐lasting antiseptic to the nares of either the infected virus‐donor guinea pig or the susceptible virus‐recipient guinea pig, or to the nares of both animals, prior to virus inoculation effectively reduced transmission of a highly transmissible influenza A virus, even when the donor and recipient guinea pigs shared the same cage. Daily treatment of the recipient guinea pig starting 1 day after initial exposure to an infected donor guinea pig in the same cage was similarly effective in preventing detectable influenza virus infection in the recipient animal.

**Conclusions:**

We conclude that a daily application of this antiseptic formulation is efficacious in reducing the transmission of influenza A virus in the guinea pig model, and further study in this and other preclinical models is warranted.

## 1 INTRODUCTION

Seasonal influenza typically causes significant global morbidity and mortality. In the United States alone, 31.4 million outpatient visits,^1^ 140,000 to 710,000 hospitalizations,^2^ and between 12,000 and 52,000 deaths^2^ result from influenza each year, with a total annual economic burden estimated in the billions of US dollars.^1^, ^3^ Influenza virus is associated with a range of symptoms, from mild upper respiratory disease characterized by fever, chills, lethargy, headache, cough, sore throat, and runny nose to severe pneumonia that may be fatal, particularly in elderly persons and in those with immunosuppressive conditions.^4^, ^5^

The main strategy for prevention and control of seasonal influenza has been vaccination. However, influenza vaccines are not optimally protective against influenza virus infection,^6^ and vaccine uptake is incomplete. In the United States, vaccination rates have not significantly changed since 2013, ranging from 41.7% in 2015–2016 to an estimated 37.1% in 2017–2018 among adults.^7^ Additionally, current influenza vaccine technologies cannot be preemptively deployed in anticipation of pandemic influenza; instead, they can be implemented only in response to a pandemic that is already underway. Nonpharmaceutical interventions (NPIs), such as mask wearing, hand hygiene, indoor ventilation, quarantine of symptomatic individuals, and social distancing for asymptomatic persons are variably effective at preventing the spread of influenza and other respiratory viruses,^8^, ^9^, ^10^ including pandemic SARS‐CoV‐2,^11^, ^12^, ^13^, ^14^ although the simultaneous implementation of multiple NPIs may be more effective than any one NPI alone.^9^, ^12^ Additional pathogen‐nonspecific interventions that are effective at hindering or preventing person‐to‐person disease transmission and that can be implemented in concert with other NPIs may help to mitigate the impact of future zoonoses like COVID‐19 or pandemic influenza.

The use of iodine as a topical antiseptic dates back nearly as far as its discovery in 1811^15^ and predates the widespread understanding of germ theory.^16^ Iodine‐based antiseptics are broadly antimicrobial; they are active against bacteria, fungi, mycobacteria, protozoa, and viruses, including influenza virus.^17^, ^18^ The inhalation of iodine vapors to treat various respiratory diseases was proposed as early as 1829,^19^ and topical, inhalational, and oral iodine preparations were used empirically to prevent influenza during the 1918 “Spanish flu”^20^, ^21^, ^22^, ^23^ and the 1957 “Asian flu”^24^ pandemics. In experiments conducted in the 1940s, the application of an ethanol‐based tincture of iodine to the snouts of mice prevented disease upon exposure to a dose of aerosolized influenza virus lethal to control mice.^25^

Subsequently, povidone‐iodine (PVP‐I) was introduced in the 1950s as a novel iodine‐based antiseptic. PVP‐I is a complex of the polymer povidone (polyvinylpyrrolidone) and triiodide (I_3_^−^) that does not require additional iodine solubilizers and is less irritating to the skin than tincture of iodine while retaining its broad‐spectrum antimicrobial activity.^17^, ^18^ Because aqueous solutions of PVP‐I maintain a low but constant concentration of free iodine (the active antimicrobial agent) in dynamic equilibrium with the PVP–triiodide complex, they are less irritating to the skin and have a shorter duration of antimicrobial activity than molecular iodine formulations like tincture of iodine or Lugol's solution.^26^, ^27^, ^28^ Thus, the use of iodine as a pathogen‐nonspecific intervention to prevent viral or bacterial infection typically entails a trade‐off between the duration of its antimicrobial activity after application and the degree to which it irritates the skin to which it is applied.

3M™ Skin and Nasal Antiseptic (PVP‐I solution 5% w/w [0.5% available iodine] USP) Patient Preoperative Skin Preparation Non‐Sterile Solution (herein after abbreviated as “Nasal Prep”) is a safe and effective formulation of 5% PVP‐I with a proprietary film‐forming composition that extends the duration of the iodine's antimicrobial activity. It has broad‐spectrum microbiocidal activity against respiratory pathogens such as *Streptococcus pneumoniae*,^29^ *Haemophilus influenzae*,^29^ methicillin‐susceptible and ‐resistant *Staphylococcus aureus*,^29^ influenza A virus,^30^ and coronaviruses,^30^ including SARS‐CoV‐2.^31^, ^32^ Clinical studies^33^, ^34^ have shown that same‐day application of Nasal Prep to the nares of preoperative patients is equivalent to or better than a 5‐day course of intranasal mupirocin at preventing postoperative surgical site infections due to *S. aureus*. In addition, polymer and excipients in the Nasal Prep formulation protect PVP‐I from inactivation by nasal mucins and other organic compounds and increase its adhesion to mucosal surfaces,^35^ potentially imparting a longer‐lasting antimicrobial effect in the nose than other PVP‐I preparations lacking these properties. We hypothesized that the PVP‐I in Nasal Prep, applied intranasally, would prevent influenza virus infection in a guinea pig model of influenza virus transmission, as had been previously shown in mice that had tincture of iodine applied to their snouts.^25^ We hypothesized further that the film‐forming property of Nasal Prep would maintain its antiviral activity over a longer duration than with standard aqueous PVP‐I solutions (typically 30–60 min),^27^ thus enabling a practicable, once‐daily reapplication interval.

## 2 METHODS

### 2.1 Virus

Influenza A/Panama/2007/1999 (H3N2) virus (Pan/99) was originally derived from a 12‐plasmid reverse genetics system.^36^ For these experiments, stock virus was subsequently propagated in Madin–Darby canine kidney (MDCK) cells stably transfected with cDNA of the human 2,6‐sialyltransferase (MDCK‐SIAT1)^37^ (Millipore Sigma/European Collection of Authenticated Cell Cultures) in Dulbecco's Modified Eagle Medium (DMEM; Gibco) supplemented with 10% fetal calf serum and 1 mg/ml of G418 selective antibiotic (Geneticin 100×, Gibco) at 37°C and 5% CO_2_.

### 2.2 Animals

Female Hartley strain guinea pigs weighing 300–350 g were obtained from Charles River Laboratories. Animals were allowed free access to food and water and kept on a 12‐h light/dark cycle. Guinea pigs were anesthetized prior to intranasal inoculation and to nasal washing, using a mixture of ketamine (30 mg/kg) and xylazine (5 mg/kg), administered intramuscularly. All procedures were performed in accordance with the Icahn School of Medicine at Mount Sinai Institutional Animal Care and Use Committee guidelines (protocol #IACUC‐2019‐0019) and were additionally approved by the US Army Medical Research and Development Command (USAMRDC) Animal Care and Use Review Office (ACURO) (protocol #DARPA‐6336). During guinea pig transmission experiments, strict measures were followed to prevent any cross‐contamination between animals, including handling recipient animals before donors and changing gloves between guinea pigs.

### 2.3 Application of nasal treatments

Nasal Prep (3M™ Skin and Nasal Antiseptic), a commercially available 5% (w/w) PVP‐I solution, was used in these studies. The vehicle comparator (Nasal Prep devoid of PVP‐I and sodium iodide) was produced by 3M specifically for these experiments, and the phosphate‐buffered saline (PBS) comparator was purchased (Gibco). All three treatments were applied to guinea pig nares in the same way: While guinea pigs were awake and in an upright position, we used a positive‐displacement pipette to deliver 50 μl to each naris (100 μl per animal). Awake administration of treatments was used instead of anesthetized administration after a comprehensive literature review^38^, ^39^, ^40^, ^41^, ^42^ and risk–benefit analysis. The viscosity of the Nasal Prep and vehicle solutions, coupled with guinea pigs' inability to protect their airways due to the loss of gag reflex while under ketamine/xylazine anesthesia, increased the risk of death by respiratory failure due to the aspiration of the solutions into the respiratory tract under anesthesia.

### 2.4 Transmission experiments

On Day 0, Pan/99 stock virus was diluted in PBS supplemented with antibiotics (Penicillin–Streptomycin 10,000 U/ml, Gibco) (PBS + P/S). An inoculum of 10^4^ plaque‐forming units (pfu) in 150 μl was instilled intranasally by applying 75 μl to each nostril. The inoculum dose was chosen to maximize the transmission rate of Pan/99 in our model. In our hands, peak nasal wash titers in donor guinea pigs reliably occur by Day 3 post inoculation at this dose (unpublished observations), and the time to peak nasal wash titer in donor guinea pigs has been shown by others to correlate inversely with transmission rate to recipients.^43^ Inoculated guinea pigs were placed supine, in a nose‐up position. Guinea pigs were then placed in the appropriate cages based on the experiment type (airborne or contact transmission). Transmission experiments were performed at constant temperature (20°C) and relative humidity (RH) (20% RH) in environmentally controlled chambers (model 6030, Caron Products & Services, Inc.). For every transmission experiment, a virus‐inoculated guinea pig donor was paired with a virus‐naïve recipient in specific cages depending on the experimental model (airborne or contact). In the contact model, each pair of animals (donor and recipient) was housed in the same cage (Figure 2A). In the airborne model, each pair of animals was housed individually in two identical transmission cages (Figure 2B), which have an open wire top and a wire‐mesh grid side panel. The transmission cages were then placed into the environmental chamber with two cages per shelf, such that the wire grids opposed each other at a separation distance of 5 cm; such an arrangement allows air to flow between cages but prevents contact between guinea pigs. Guinea pigs were kept together for a total of 7 days. Nasal washing was performed on Days 1, 3, 5, and 7 post inoculations by instilling a total of 1 ml of PBS + P/S into both nares and allowing it to drain onto a sterile Petri dish. Nasal treatments were reapplied every 24 h. Nasal wash samples were collected in 1.5‐ml tubes on ice, centrifuged to pellet debris, and stored at −80°C until titration by plaque assay, as previously described.^44^

### 2.5 Experimental design and statistical analysis

These exploratory experiments were performed in the absence of existing data to inform an estimate for effect size; thus, power calculations were not carried out a priori. Transmission experiments were performed in groups of four or eight transmission pairs (eight or 16 guinea pigs) in one or two transmission chambers at a time. Experiments were designed sequentially, with prior results informing the next experimental scheme to be tested, and the results are presented in the order in which experiments were conducted. Given the exploratory nature of these experiments, not every possible combination of intranasal treatment (Nasal Prep, vehicle, or PBS), treated animal (inoculated virus donor, susceptible virus recipient, or both), or transmission model (contact or airborne) was tested. Where possible, we minimized animal use by omitting groups that were unlikely to be substantively additionally informative, given prior results; the rationales for these omissions are noted in the text describing each experiment. Because power calculations were not performed and group sizes not established a priori, Bayesian methods^45^ were used to evaluate the strength of the evidence in favor of the null hypothesis (the virus transmission rates are not different in different treatment groups) or its alternative (the transmission rates are different between different treatment groups). Transmission experiment results were tabulated in 2 × 2 matrices, with treatment group in rows and transmission (yes or no) as columns. Bayes factors and posterior probabilities were calculated in R^46^, ^47^ with the package *BayesFactor* (v.0.9.12–4.2)^45^, ^48^, ^49^ (Text S1). Because the result of interest is the proportion of successful transmission events in each treatment group, the Bayes factor for independent multinomial sampling was calculated as a test for equality of proportions, with the row (treatment) margins fixed.^45^ The magnitude of the Bayes factor quantifies the strength of evidence in support of the alternative hypothesis over the null hypothesis. For example, a Bayes factor of 5 means that, given the data observed, the alternative hypothesis is five times more likely to be correct than the null hypothesis, while a Bayes factor of 0.2 (the reciprocal of 5) indicates that the alternative hypothesis is five times *less* likely than the null hypothesis.^45^ Bayes factors, being essentially odds ratios, can alternatively be expressed as the percent likelihood, from 0% to 100%, that a given hypothesis is likely to be the correct one, given the observed data (i.e., the posterior probability).^48^

## 3 RESULTS

**FIGURE 1:**
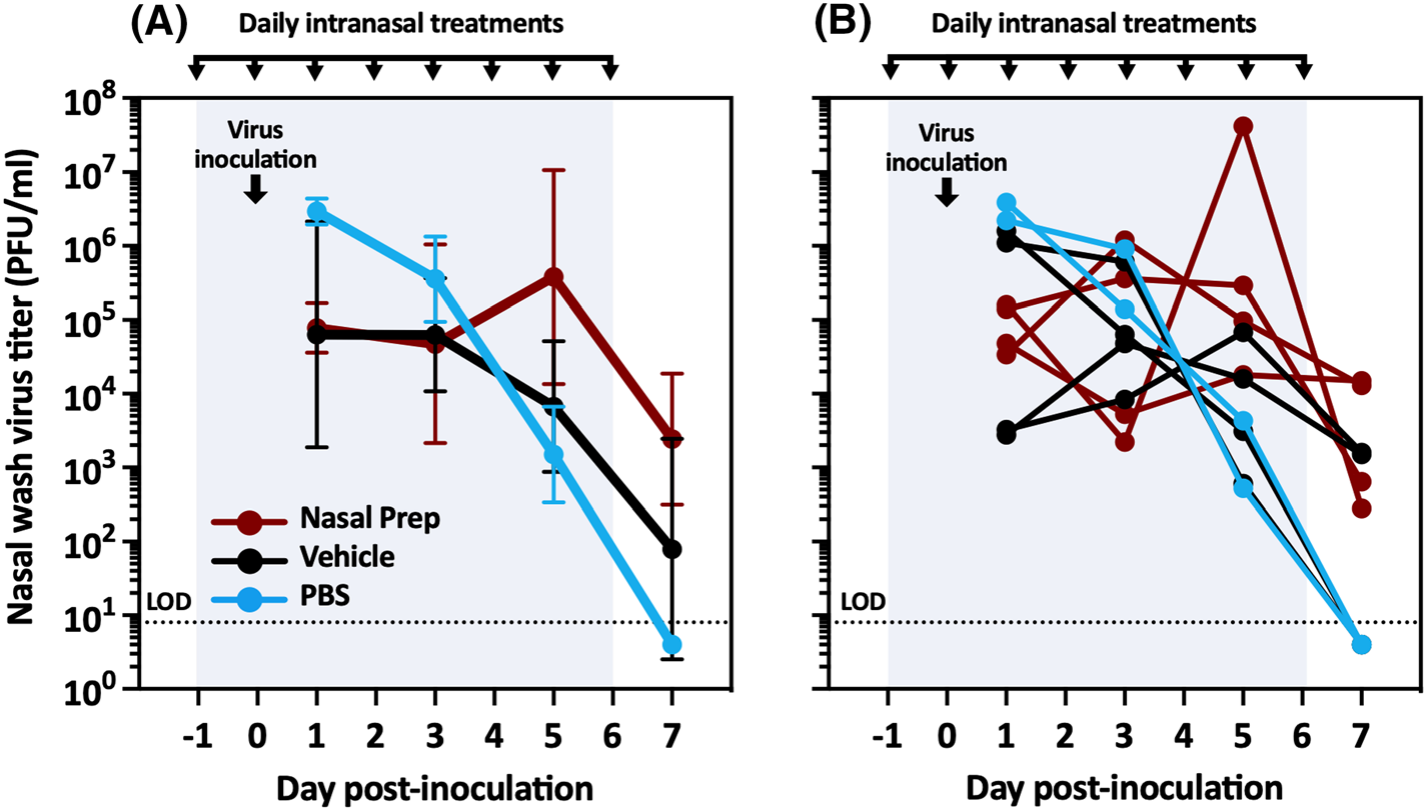
Influenza virus replication in directly virus‐inoculated and treated guinea pigs. (A) Summary data (geometric mean and geometric standard deviation) of nasal wash virus titers in directly inoculated guinea pigs treated with daily application of Nasal Prep, its vehicle, or PBS, starting 1 day prior to inoculation. (B) Nasal wash virus titers of the individual guinea pigs summarized in panel (A). LOD, limit of detection

To assess the effect of topical Nasal Prep applied prophylactically in the nares of guinea pigs intranasally inoculated with influenza virus, we compared Nasal Prep to two controls, the Nasal Prep “vehicle” (Nasal Prep devoid of PVP‐I and other iodinated species) or PBS. Four guinea pigs were treated intranasally with Nasal Prep, four with the vehicle, and two with PBS. Twenty‐four hours after treatment, all 10 guinea pigs were inoculated intranasally with influenza A/Panama/2007/1999 (H3N2) (“Pan/99”). All guinea pigs underwent nasal washes for virus titration every 2 days, starting on Day 1 post inoculation. The treatments (Nasal Prep, vehicle, or PBS) were also reapplied every 24 h; on nasal wash days, the treatment reapplication was done after nasal washing. As shown in Figure 1A, nasal wash virus titers generally decreased over time, consistent with an intact innate immune response. The PBS‐treated guinea pigs demonstrated higher titers immediately after inoculation, which decreased steadily to undetectable levels from Day 1 to Day 7 post inoculation, and the viral loads in the two animals were similar throughout the course of infection (Figure 1B). The Nasal Prep‐ and vehicle‐treated groups, however, displayed more variable titers between animals and from day to day (Figure 1B), suggesting incomplete neutralization of directly inoculated influenza virus by Nasal Prep. Interestingly, the nasal wash virus titers of the vehicle‐treated animals were intermediate between the PBS control and Nasal Prep groups, indicating an effect of the vehicle alone on influenza virus replication in vivo.

Even though the use of Nasal Prep did not result in consistent reduction of viral titers in the nasal washes of directly inoculated guinea pigs, the infectious dose required to infect a virus‐naïve, susceptible guinea pig (the “virus recipient”) via animal‐to‐animal transmission from an inoculated guinea pig (the “virus donor”) is likely to be orders of magnitude lower than 10^4^ pfu, the inoculum dose in the prior experiment. We^36^ and others^43^, ^50^ have published data that cumulatively suggest that the median infectious dose of Pan/99 is on the order of 10^1^ pfu; thus, we proceeded to explore whether Nasal Prep could reduce influenza virus transmission in the guinea pig model.

**FIGURE 2:**
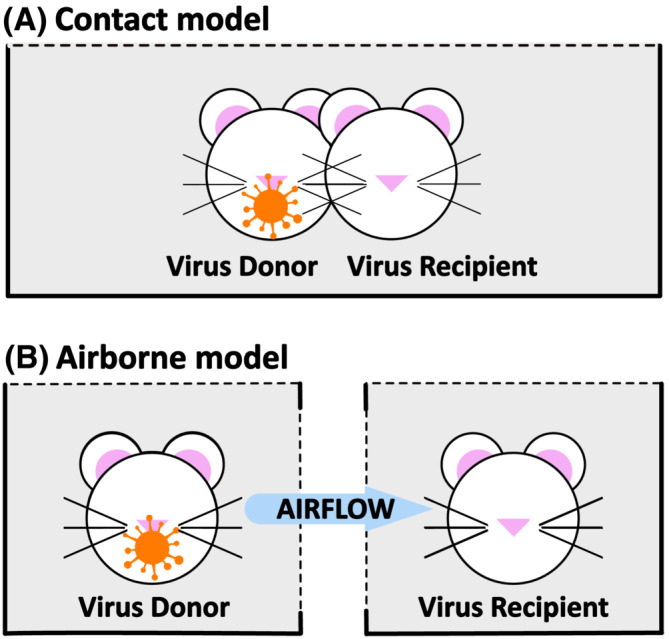
Transmission models. Schematics of (A) contact and (B) airborne models of influenza virus transmission in guinea pigs. The orange virus symbol represents the virus‐inoculated donor guinea pig.

In the guinea pig influenza virus transmission model, guinea pigs can transmit virus between one another while occupying the same or separate cages. In the contact transmission model (Figure 2A), both virus‐donor and ‐recipient guinea pigs are in the same cage; thus, all routes of transmission, both contact and airborne, are available to the virus. In the airborne model (Figure 2B), virus‐donor and ‐recipient guinea pigs are separated by an air‐permeable partition that precludes physical contact between the guinea pigs or their environments. Physical separation of donor and recipient necessitates that virus transmission occurs via an airborne route and over a larger distance between donor and recipient, representing a more stringent barrier to transmission that the virus must overcome. Conversely, then, the contact model presents a more stringent challenge to an intervention deployed to prevent transmission, because it presents less of a barrier to transmission than the airborne model.

**FIGURE 3:**
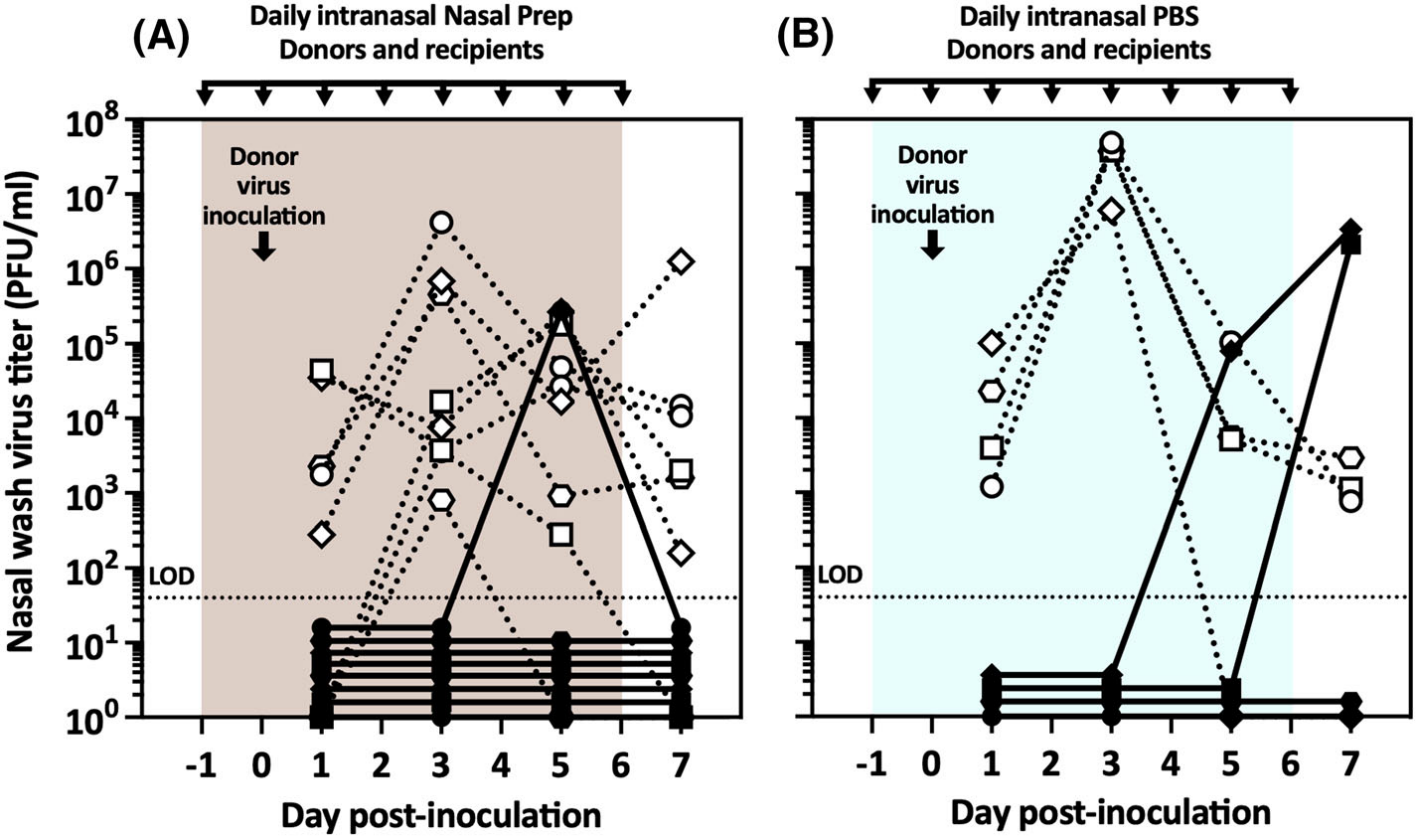
Preinoculation treatment of both donor and recipient guinea pigs in the airborne transmission model. Nasal wash virus titers of donor and recipient guinea pigs treated with daily intranasal (A) Nasal Prep or (B) PBS from Day −1 to Day 6 post inoculation. Dotted lines/open symbols represent nasal wash virus titers of donor animals; solid lines/closed symbols represent nasal wash virus titers of recipient animals. LOD, limit of detection

We first tested the efficacy of Nasal Prep in preventing airborne transmission of influenza virus in the guinea pig model. Twenty‐four hours before infecting the donor guinea pigs, we applied Nasal Prep or PBS to all animals' nares, both donors and recipients. The next day, donor guinea pigs were inoculated with Pan/99, and then, 3 h later, the inoculated virus‐donor animals and susceptible virus‐recipient animals were retreated with Nasal Prep or PBS and then paired in cages that only permit virus transmission via the airborne route (as shown in Figure 2B). Intranasal treatments were reapplied every 24 h throughout the experiment, and nasal washes for virus titration were performed every other day. Two experimental replicates (each with four donor–recipient guinea pig pairs) were performed with Nasal Prep to confirm reproducibility of results, and one replicate was performed with PBS. As we had seen previously in the direct inoculation experiment (Figure 1), PBS treatment of donor guinea pigs had little effect on the nasal wash virus titers, compared with historical data obtained in untreated guinea pigs inoculated with Pan/99 (e.g., previous studies^36^, ^43^, ^50^); however, the Nasal Prep‐treated donors displayed much more variability in nasal wash titers over the course of the experiment (Figure 3). In the Nasal Prep‐treated group, we observed influenza virus transmission in one pair out of eight (12.5%) with only one positive nasal wash, on Day 5 post inoculation; the subsequent nasal wash on Day 7 did not yield titratable virus in plaque assay. In comparison, transmission was observed in two pairs out of four (50%) treated with PBS (Bayes factor = 1.32). This Bayes factor indicates that, given the observed data, the alternative hypothesis is only slightly favored over the null: There is a 57% likelihood that the transmission rates are truly different between Nasal Prep‐ and PBS‐treated guinea pigs and, correspondingly, a 43% likelihood that they are not truly different; thus, these data provide insufficient evidence to meaningfully support either hypothesis over the other.^45^ In the airborne model, Pan/99 is typically transmitted from untreated donors to 75% to 100% of untreated recipients^4^, ^51^; thus, while the 50% transmission rate in the PBS group is lower than would be expected if PBS treatment of both animals had no effect on transmission, the small group size provides insufficient evidence to conclusively reject the null hypothesis (Bayes factor = 2.84, which corresponds to a 26% likelihood that the transmission rate obtained in the PBS group is not actually different from the rate historically observed with this virus in untreated guinea pigs). However, Nasal Prep treatment of both donors and recipients did markedly decrease transmission below what would be expected in untreated guinea pigs^4^, ^51^ (Bayes factor = 2949; >99% likelihood that the transmission rate observed in this group is truly different from the historical data). Thus, to reduce animal use, we chose not perform any further replicates in which both donor and recipient are treated, but rather to proceed directly to transmission experiments in which only one animal in each transmission pair is treated.

**FIGURE 4:**
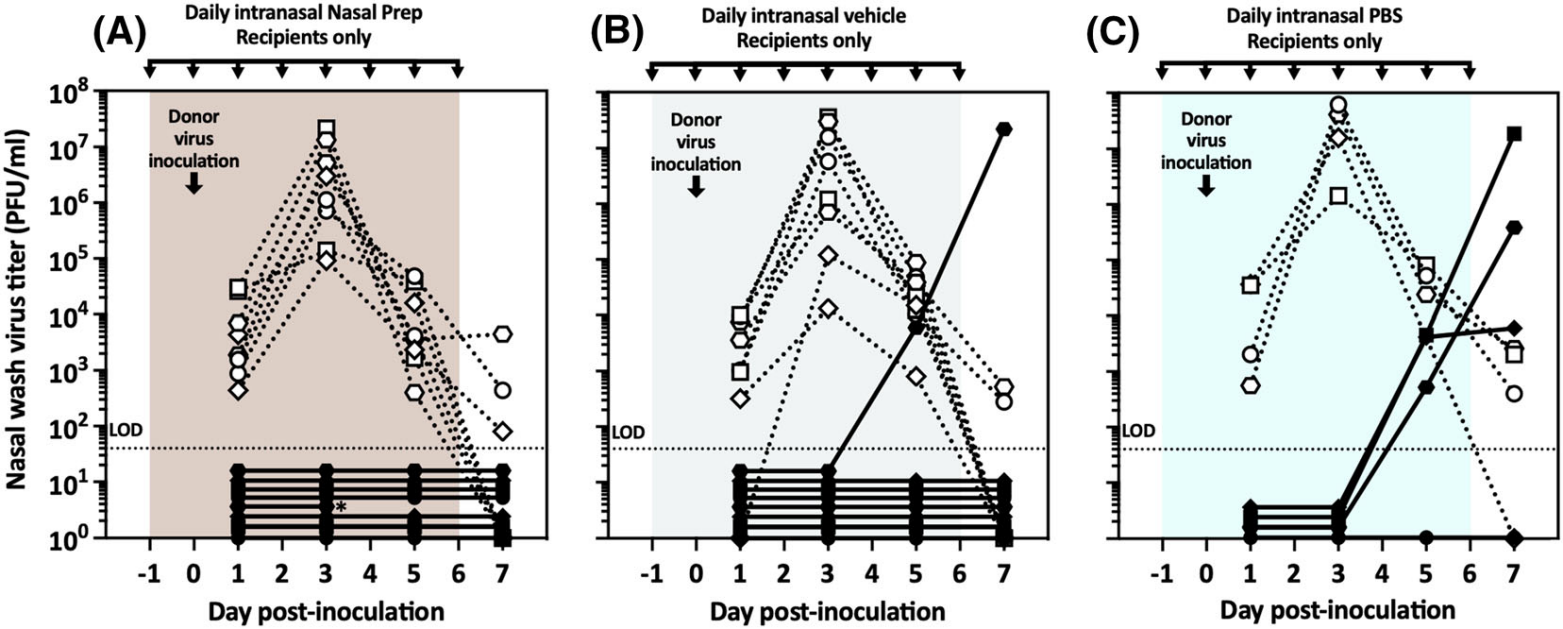
Pre‐exposure treatment of only recipient guinea pigs in the airborne transmission model. (A) No transmission events were observed in recipient guinea pigs treated with Nasal Prep. One recipient (*) died under anesthesia following nasal wash on Day 3; that pair was not included in the overall transmission rate (zero of seven pairs). (B) One transmission event was observed in eight recipients treated with vehicle (12.5%). (C) Three of four (75%) of recipients treated with intranasal PBS became infected by virus transmission from untreated donor guinea pigs. Dotted lines/open symbols represent nasal wash virus titers of donor animals; solid lines/closed symbols represent nasal wash virus titers of recipient animals. LOD, limit of detection

To model a prophylactic intervention, in which intranasal treatments would be applied to prevent the user from influenza virus infection, and to determine whether treatment of infected, susceptible, or both animals was necessary to prevent transmission between them, we next treated only recipient guinea pigs with Nasal Prep, its vehicle (Nasal Prep devoid of any iodine), or PBS, starting 24 h prior to virus inoculation of untreated donor guinea pigs. This experiment was performed using the airborne transmission model in the same way as the prior one (Figure 3). As expected, the nasal washes of the untreated donor guinea pigs displayed similar virus titers over time as those from PBS‐treated donors (Figure 3C), and similar also to historical data in untreated guinea pigs (e.g., previous studies^36^, ^43^, ^50^). Daily intranasal application of Nasal Prep to only the recipient guinea pigs prevented influenza virus transmission in all seven donor–recipient pairs (0%; Figure 4A), while PBS treatment did not prevent virus transmission to three of four recipient guinea pigs (75%; Figure 4C) (Bayes factor = 12.4, which corresponds to a 93% likelihood that the transmission rates are truly different between Nasal Prep‐ and PBS‐treated groups^45^). Vehicle treatment of recipient guinea pigs also prevented virus transmission in all but one transmission pair (transmission rate 12.5%; Figure 4B), likely similar to the Nasal Prep‐treated group (Bayes factor = 0.42, which corresponds to a 71% likelihood that the transmission rates are not actually different between Nasal Prep‐ and vehicle‐treated groups), but different from the PBS‐treated group (Bayes factor = 4.46; 82% likelihood that the transmission rates are truly different between vehicle‐ and PBS‐treated groups^45^). We performed two replicates (four donor–recipient guinea pig pairs per replicate) with Nasal Prep and its vehicle to ensure reproducibility of results. We performed only one replicate with PBS to reduce animal use; historically, a 75% transmission rate (three of four untreated guinea pig pairs) would be an expected result with this virus in this model.^4^, ^51^

**FIGURE 5:**
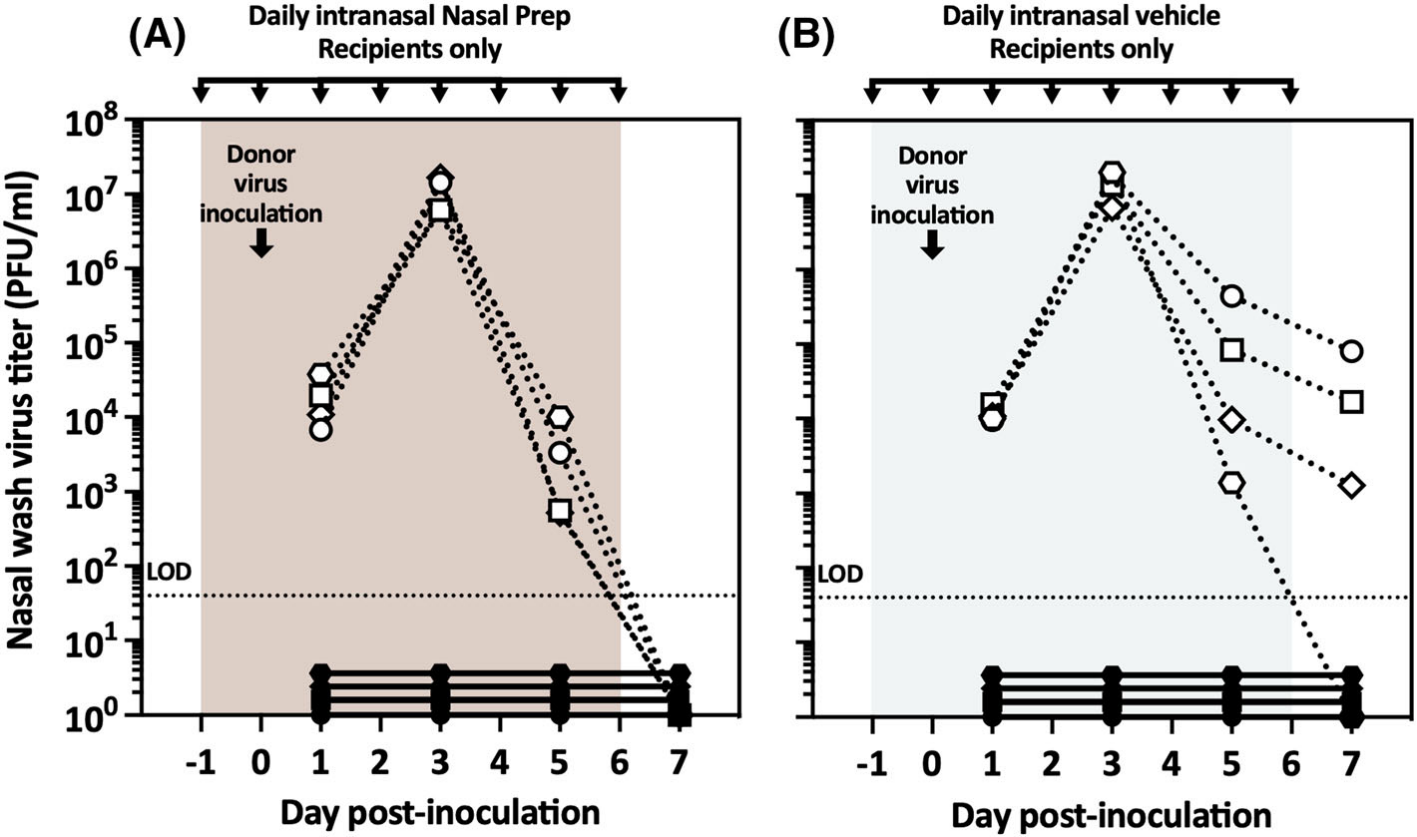
Pre‐exposure treatment of only recipient guinea pigs in the contact transmission model. (A) No transmission events were observed in four recipient guinea pigs treated with nasal prep. (B) No transmission events were observed in four recipients treated with vehicle. Dotted lines/open symbols represent nasal wash virus titers of donor animals; solid lines/closed symbols represent nasal wash virus titers of recipient animals. LOD, limit of detection

Because daily intranasal Nasal Prep efficiently prevented influenza virus transmission to treated recipient guinea pigs in the airborne model, we next evaluated its efficacy in the contact model (as shown in Figure 2A), which presents a lower barrier to virus transmission and thus a more stringent challenge to Nasal Prep as a prophylactic intervention to protect against recipient infection in this model. In this experiment, we compared Nasal Prep and vehicle treatment of recipient guinea pigs housed in the same cage as their untreated, infected donor guinea pig partner (Figure 5). To reduce animal use, we did not include a PBS control group because it had been relatively ineffective at preventing transmission in the airborne model and thus would be expected to be equally as or even more ineffective in a contact model.^4^, ^51^ This experiment otherwise followed the same protocol as prior ones (Figures 3 and 4). Both Nasal Prep (Figure 5A) and its iodine‐free vehicle (Figure 5B) prevented influenza virus transmission to all four treated recipient guinea pigs.

**FIGURE 6:**
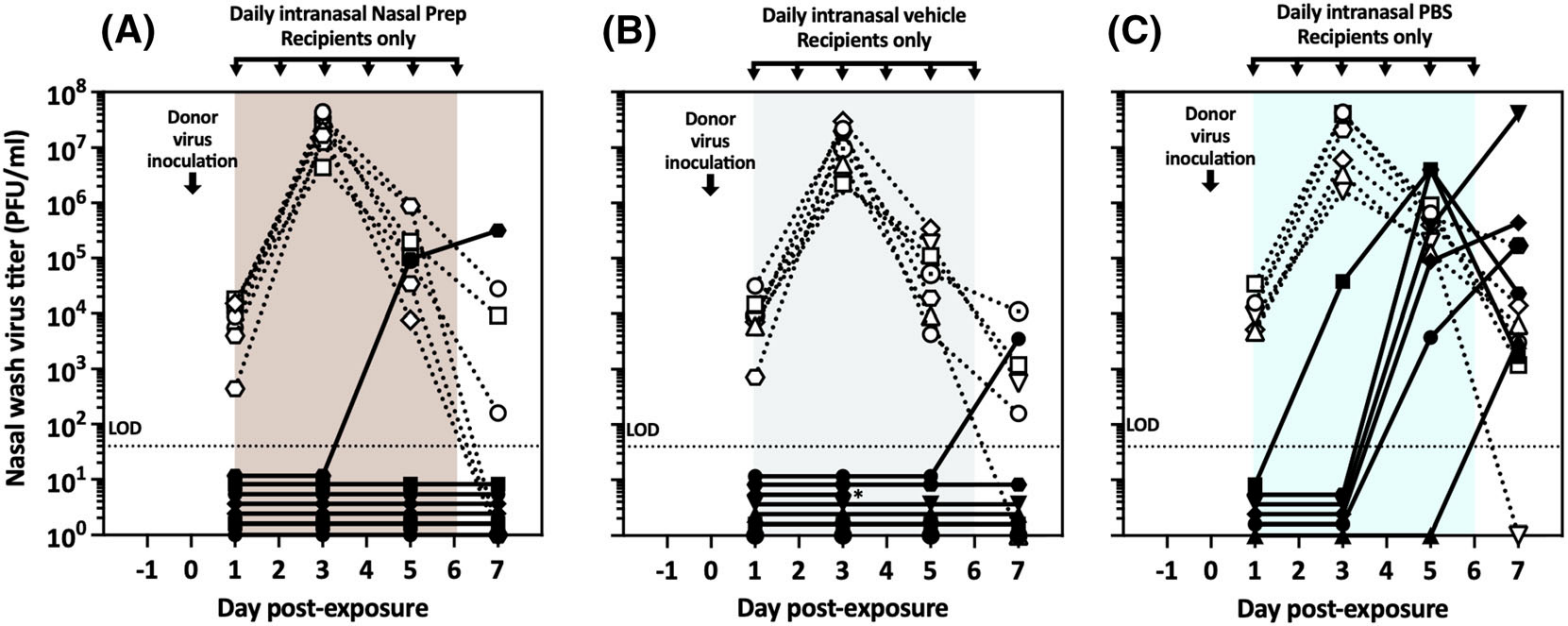
Postexposure prophylaxis of virus‐recipient guinea pigs in the contact transmission model. (A) Transmission was observed in one of seven recipient guinea pigs (14.3%) treated with nasal prep starting 1 day after exposure to infected donor guinea pigs. (B) Transmission was observed in one of seven recipients treated with vehicle starting 1 day after exposure to infected donors. One recipient (*) died under anesthesia following nasal wash on Day 3; that pair was not included in the calculations of the overall transmission rate (one of six pairs, 16.7%). (C) Six of six (100%) of recipients treated with intranasal PBS became infected by virus transmission from untreated donor guinea pigs. Dotted lines/open symbols represent nasal wash virus titers of donor animals; solid lines/closed symbols represent nasal wash virus titers of recipient animals. LOD, limit of detection

We next assessed the effectiveness of the topical intranasal treatments to prevent influenza virus transmission to recipient guinea pigs when used as postexposure prophylaxis (PEP), with treatment application starting 1 day after the recipient guinea pigs had already been cocaged with untreated, infected donor guinea pigs. In this experiment, donor guinea pigs were intranasally inoculated with influenza virus under anesthesia. Three hours later, after the donor animals had fully recovered from anesthesia, each donor was paired with an untreated recipient guinea pig in the same cage (contact transmission model, Figure 2A). One day post inoculation (21 h post exposure of untreated recipients to infected donors), nasal washes were performed on all guinea pigs for virus titration, and then, all recipient guinea pigs received an initial application of Nasal Prep, vehicle, or PBS. As with all prior experiments, intranasal treatments were subsequently reapplied every 24 h throughout the experiment, and nasal washes for virus titration were performed every other day (Figure 6). One experimental replicate (four donor–recipient guinea pig pairs) was performed with each of the three treatments, and then, a second replicate was performed with Nasal Prep (three pairs), vehicle (three pairs), and PBS (two pairs), for a total of eight donor–recipient pairs in the replicate group, which is the maximum number of transmission pairs that can be accommodated in parallel in our facilities. Both Nasal Prep and vehicle, applied as PEP, efficiently blocked virus transmission in the contact model, with one transmission event in each group (Bayes factor = 0.46; 68% likelihood that the transmission rates are not actually different between Nasal Prep‐ and vehicle‐treated groups). In comparison with historical data with Pan/99 in untreated guinea pigs in the contact model,^4^, ^51^ intranasal PBS had no measurable effect on virus transmission, with all six PBS‐treated recipient guinea pigs becoming infected by transmission from a co‐caged donor animal (PBS vs. Nasal Prep, Bayes factor = 61.3; 98% likelihood that the transmission rates are truly different between PBS‐ and Nasal Prep‐treated groups, and PBS vs. vehicle, Bayes factor = 35.0; 97% likelihood that the transmission rates are truly different between PBS‐ and vehicle‐treated groups).

**FIGURE 7:**
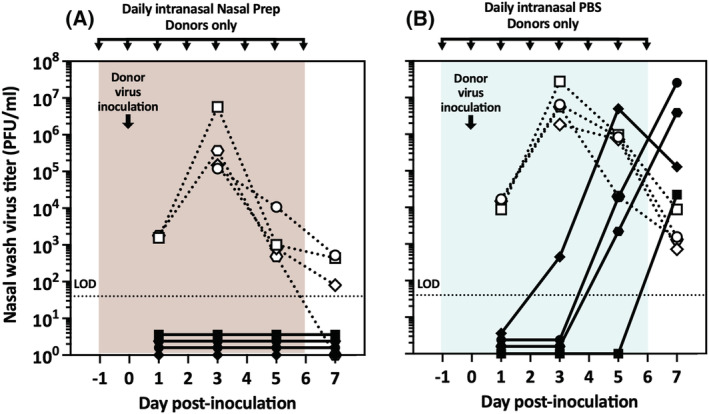
Preinoculation treatment of only donor guinea pigs in the contact transmission model. (A) No transmission was observed in four untreated recipient guinea pigs exposed to infected, treated donor guinea pigs. (B) Four transmission events were observed in four recipients exposed to infected, treated donor guinea pigs. Dotted lines/open symbols represent nasal wash virus titers of donor animals; solid lines/closed symbols represent nasal wash virus titers of recipient animals. LOD, limit of detection

Finally, we performed an experiment to determine the effect of intranasal treatment of donor guinea pigs on transmission to untreated recipient guinea pigs in the contact model. We applied Nasal Prep or PBS intranasally in donor guinea pigs 24 h before inoculating them with influenza virus. Treatments were reapplied 3 h after inoculation, and then infected, treated donors were cocaged with untreated recipient guinea pigs. Thereafter, as in prior experiments, intranasal treatments were reapplied every 24 h throughout the experiment, and nasal washes for virus titration were performed every other day (Figure 7). One experimental replicate (four donor–recipient guinea pig pairs) was performed with Nasal Prep and one with PBS. A vehicle group was not performed and the Nasal Prep and PBS groups were not replicated because the contract funding period expired concurrent with this experiment. Nasal Prep treatment of donor guinea pigs prevented influenza virus transmission to recipient guinea pigs, while PBS treatment of donors did not (0% vs. 100% transmission, Bayes factor = 25.2; 96% likelihood that the transmission rates are truly different between Nasal Prep‐ and PBS‐treated groups). In this replicate, the Nasal Prep‐treated donors displayed less animal‐to‐animal variation in viral load, compared with prior experiments with Nasal Prep‐treated donors (Figures 1 and 3), and their nasal wash titer curves appear more similar in shape, though not in magnitude, to those of the PBS‐treated donors in the same experiment. In fact, all nasal wash titers from the Nasal Prep‐treated donors in Figure 7A except for two (one animal on Day 3 and one animal on Day 7 post inoculation) fall within one geometric standard deviation of the geometric mean nasal wash titers of the 12 Nasal Prep‐treated donor guinea pigs presented in Figures 1 and 3 (Figure S1). That the curves happen to look similar to each other is, we speculate, due to random chance in a small number of animals.

## 4 DISCUSSION

In these experiments, we observed that daily intranasal application of Nasal Prep impedes the transmission of an influenza A virus in the guinea pig model, even when inoculated virus‐donor and susceptible virus‐recipient guinea pigs are housed in the same cage. Further, Nasal Prep was efficacious in reducing the frequency of virus transmission to recipient guinea pigs when the treatment was applied to virus recipients up to 24 h after they were exposed to infected donor guinea pigs in the same cage. These results, though preliminary, indicate that further investigation of Nasal Prep as a broad‐spectrum intranasal antiseptic to prevent the transmission of influenza and other respiratory viruses is warranted.

Interestingly, the vehicle alone, without PVP‐I, also reduced influenza virus transmission in our model. The mechanism remains to be elucidated, but hypotheses include its pH being unfavorable for virus infectivity and/or its viscosity physically impeding the virus particle from reaching or interacting with host cells. Others have demonstrated that reducing the burden of Gram‐positive bacterial nasal flora by preinoculation mupirocin decolonization impairs the transmissibility of influenza virus in ferrets,^52^ which may also be contributing to the relative inefficiency of influenza virus transmission in Nasal Prep‐ and perhaps also vehicle‐treated guinea pigs. The number of transmission pairs in these experiments lacks sufficient statistical power to discern whether PVP‐I, which has been shown to be virucidal for influenza viruses,^18^ confers an additive benefit to the Nasal Prep vehicle alone in preventing influenza virus transmission in this model. It is, however, well known that enveloped viruses, such as influenza virus, are significantly easier to inactivate than non‐enveloped viruses and many bacteria.^53^ Thus, in situations such as the emergence of a new pandemic pathogen yet to be identified or characterized, the broader spectrum of PVP‐I‐containing Nasal Prep could be indispensable; however, further investigation is required to establish the role of Nasal Prep in preventing the spread of other respiratory viruses and bacteria. Finally, the antibacterial activity of Nasal Prep could potentially provide a benefit in preventing postinfluenza secondary bacterial infections that arise from intranasal colonizing flora like *S. pneumoniae* and *S. aureus*,^54^ but this hypothesis also needs further study.

Further investigation is also necessary to determine the safety and tolerability of long‐term use of Nasal Prep. Although no overt signs of discomfort were observed in guinea pigs, the duration of treatment in these experiments (a maximum of eight daily applications) is insufficient to establish that Nasal Prep can be used on a daily basis for longer periods of time, such as during a respiratory virus epidemic or pandemic. Systemic absorption of elemental iodine from mucous membranes is approximately 5–10 times greater than from the intact skin.^55^ Most people tolerate excess iodine absorption without significant physiological consequences, with important factors being the duration, dose, and frequency of iodine exposure and the underlying kidney and thyroid function of the individual^55^; these factors must be studied in greater depth before this intervention could be deployed in humans. Despite these potential limitations, these experiments provide a strong rationale for further investigation of Nasal Prep as a pathogen‐nonspecific intervention that may reduce the risk of respiratory virus infection including, potentially, future zoonotic viruses that have yet to manifest in humans.

## CONFLICT OF INTEREST

Dr. Bouvier reports that she is an employee of the Icahn School of Medicine at Mount Sinai, which received funding from 3M Company during the conduct of the study. Dr. Landgrebe, Dr. Parthasarathy, and Dr. Wlashin report that they are employees of 3M, which received funding from DARPA during the conduct of the study. 3M employees conceived of the study and contributed to experimental design but had no direct participation in the conduct of the experiments or the collection or analysis of the data. Dr. Landgrebe, Dr. Parthasarathy, and Dr. Wlashin also report three patents pending that are relevant to this work, entitled “Treatment and Prophylaxis of Viral Infections,” “Transmission Prevention of Viruses with Application of Antiseptic Composition,” and “Virus Transmission with Application of a Composition.” Dr. Gaaloul and Mr. Komen report having nothing to disclose.

## AUTHOR CONTRIBUTIONS

**Nassima Gaaloul ben Hnia:** Investigation; methodology. **Mathew Kipkemboi Komen:** Investigation; methodology. **Katie F. Wlaschin:** Conceptualization; funding acquisition; resources. **Ranjani V. Parthasarathy:** Conceptualization; funding acquisition; resources. **Kevin D. Landgrebe:** Conceptualization; funding acquisition; resources.

### PEER REVIEW

The peer review history for this article is available at https://publons.com/publon/10.1111/irv.13035.

## Supporting information


**Figure S1.** Comparison of nasal wash titers from Nasal Prep‐treated donor guinea pigs presented in Figure 7 to those presented in Figure 1 and Figure 3. The geometric mean (brown closed symbols and solid lines) and standard deviation (brown dashed lines) of the nasal wash virus titers from the 12 Nasal Prep‐treated donor guinea pigs shown in Figures 1 and 3A are presented with the individual nasal wash titers of the 4 Nasal Prep‐treated guinea pigs shown in Figure 7A (red open symbols and dotted lines). The horizontal black dotted line represents the limit of detection (LOD) of the plaque assay; points plotted below the LOD are graphed at y = 1 to allow presentation on a log‐scale axis.
**Text S1.** R Script and Console Output for Bayes Factor and Posterior Probability Calculations

## Data Availability

The data that support the findings of this study are available from the corresponding author upon reasonable request.
