# Supplementary material for: Intranasal antisepsis to reduce influenza virus transmission in an animal model
Source: Influenza Other Respir Viruses. 2022 Oct 12;17(1):e13035. doi: 10.1111/irv.13035 (PMC9835424; doi:10.1111/irv.13035)
Supplement: Supplementary file 1 — Figure S1. Comparison of nasal wash titers from Nasal Prep‐treated donor guinea pigs presented in Figure 7 to those presented in Figure 1 and Figure 3. The geometric mean (brown closed symbols and solid lines) and standard deviation (brown dashed lines) of the nasal wash virus titers from the 12 Nasal Prep‐treated donor guinea pigs shown in Figures 1 and 3A are presented with the individual nasal wash titers of the 4 Nasal Prep‐treated guinea pigs shown in Figure 7A (red open symbols and dotted lines). The horizontal black dotted line represents the limit of detection (LOD) of the plaque assay; points plotted below the LOD are graphed at y = 1 to allow presentation on a log‐scale axis. Text S1. R Script and Console Output for Bayes Factor and Posterior Probability Calculations [file IRV-17-e13035-s001.docx]

**Supplementary Information for**

***Intranasal Antisepsis to Reduce Influenza Virus Transmission in an Animal Model***

Nassima Gaaloul ben Hnia, Mathew Kipkemboi Komen, Katie F. Wlaschin,

Ranjani V. Parthasarathy, Kevin D. Landgrebe, Nicole M. Bouvier

___________________________________________________________________________

**
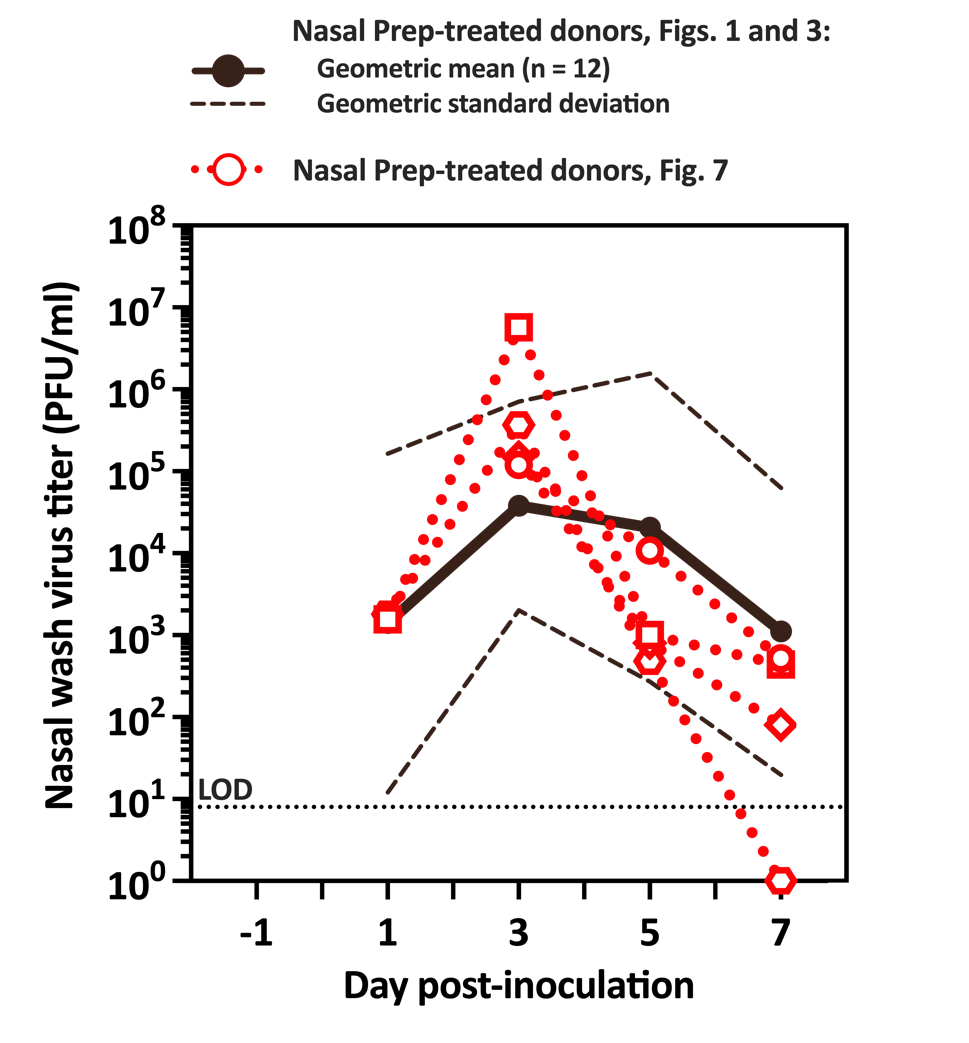
**

**Supplementary Figure 1. *Comparison of nasal wash titers from Nasal Prep-treated donor guinea pigs presented in Figure 7 to those presented in Figure 1 and Figure 3.*** The geometric mean (brown closed symbols and solid lines) and standard deviation (brown dashed lines) of the nasal wash virus titers from the 12 Nasal Prep-treated donor guinea pigs shown in Figs. 1 and 3A are presented with the individual nasal wash titers of the 4 Nasal Prep-treated guinea pigs shown in Fig. 7A (red open symbols and dotted lines). The horizontal black dotted line represents the limit of detection (LOD) of the plaque assay; points plotted below the LOD are graphed at y=1 to allow presentation on a log-scale axis.

**Supplementary Text 1. *R Script and Console Output for Bayes Factor and Posterior Probability Calculations***

- **R script**

library(BayesFactor)

# Read in experimental results from an Excel file.

fileName <- fileName <- "Data.csv"

Data <- read.csv(fileName, header = TRUE, sep = ",")

# Format experimental results as 2x2 matrices (contingency tables).

# Matrices named as follows:

# dataX => data in Figure X

# dataXNvV => 2x2 matrix, Nasal Prep vs. Vehicle, from Figure X

# dataXNvP => 2x2 matrix, Nasal Prep vs. PBS, from Figure X

# dataXVvP => 2x2 matrix, Vehicle vs. PBS, from Figure X

data3 <- matrix(data = Data[ ,3], nrow = 3, ncol = 2, byrow = FALSE,

dimnames = list(c("Nasal Prep","Vehicle", "PBS"),

c("Transmission","No transmission")))

data3 <- data3[-2, ]

data4 <- matrix(data = Data[ ,4], nrow = 3, ncol = 2, byrow = FALSE,

dimnames = list(c("Nasal Prep","Vehicle", "PBS"),

c("Transmission","No transmission")))

data4NvV <- data4[-3, ]

data4NvP <- data4[-2, ]

data4VvP <- data4[-1, ]

data5 <- matrix(data = Data[ ,5], nrow = 3, ncol = 2, byrow = FALSE,

dimnames = list(c("Nasal Prep","Vehicle", "PBS"),

c("Transmission","No transmission")))

data5 <- data5[-3, ]

data6 <- matrix(data = Data[ ,6], nrow = 3, ncol = 2, byrow = FALSE,

dimnames = list(c("Nasal Prep","Vehicle", "PBS"),

c("Transmission","No transmission")))

data6NvV <- data6[-3, ]

data6NvP <- data6[-2, ]

data6VvP <- data6[-1, ]

data7 <- matrix(data = Data[ ,7], nrow = 3, ncol = 2, byrow = FALSE,

dimnames = list(c("Nasal Prep","Vehicle", "PBS"),

c("Transmission","No transmission")))

data7 <- data7[-2, ]

# Calculate Bayes factors for all 2x2 contingency tables [1].

# sampleType = "indepMulti" => Independent multinomial sampling scheme

# fixedMargin = "rows" => Row (treatment) marginal totals are fixed, and,

# consequently, cell counts are multinomially distributed within each row.

# For a 2 × 2 contingency table, the Bayes factor for the independent multinomial

# sampling plan reduces to a test for the equality of two proportions.

BFfig3 <- contingencyTableBF(data3,sampleType = "indepMulti", fixedMargin = "rows")

BFfig5 <- contingencyTableBF(data5,sampleType = "indepMulti", fixedMargin = "rows")

BFfig7 <- contingencyTableBF(data7,sampleType = "indepMulti", fixedMargin = "rows")

BFfig4NvV <- contingencyTableBF(data4NvV,sampleType = "indepMulti", fixedMargin = "rows")

BFfig4NvP <- contingencyTableBF(data4NvP,sampleType = "indepMulti", fixedMargin = "rows")

BFfig4VvP <- contingencyTableBF(data4VvP,sampleType = "indepMulti", fixedMargin = "rows")

BFfig6NvV <- contingencyTableBF(data6NvV,sampleType = "indepMulti", fixedMargin = "rows")

BFfig6NvP <- contingencyTableBF(data6NvP,sampleType = "indepMulti", fixedMargin = "rows")

BFfig6VvP <- contingencyTableBF(data6VvP,sampleType = "indepMulti", fixedMargin = "rows")

# Convert Bayes factor to a % likelihood [2].

priorOddsFig3 <- newPriorOdds(BFfig3, type = "equal")

postOddsFig3 <- priorOddsFig3 * BFfig3

postProbFig3 <- as.BFprobability(postOddsFig3)

priorOddsFig4NvV <- newPriorOdds(BFfig4NvV, type = "equal")

postOddsFig4NvV <- priorOddsFig4NvV * BFfig4NvV

postProbFig4NvV <- as.BFprobability(postOddsFig4NvV)

priorOddsFig4NvP <- newPriorOdds(BFfig4NvP, type = "equal")

postOddsFig4NvP <- priorOddsFig4NvP * BFfig4NvP

postProbFig4NvP <- as.BFprobability(postOddsFig4NvP)

priorOddsFig4VvP <- newPriorOdds(BFfig4VvP, type = "equal")

postOddsFig4VvP <- priorOddsFig4VvP * BFfig4VvP

postProbFig4VvP <- as.BFprobability(postOddsFig4VvP)

priorOddsFig5 <- newPriorOdds(BFfig5, type = "equal")

postOddsFig5 <- priorOddsFig5 * BFfig5

postProbFig5 <- as.BFprobability(postOddsFig5)

priorOddsFig6NvV <- newPriorOdds(BFfig6NvV, type = "equal")

postOddsFig6NvV <- priorOddsFig6NvV * BFfig6NvV

postProbFig6NvV <- as.BFprobability(postOddsFig6NvV)

priorOddsFig6NvP <- newPriorOdds(BFfig6NvP, type = "equal")

postOddsFig6NvP <- priorOddsFig6NvP * BFfig6NvP

postProbFig6NvP <- as.BFprobability(postOddsFig6NvP)

priorOddsFig6VvP <- newPriorOdds(BFfig6VvP, type = "equal")

postOddsFig6VvP <- priorOddsFig6VvP * BFfig6VvP

postProbFig6VvP <- as.BFprobability(postOddsFig6VvP)

priorOddsFig7 <- newPriorOdds(BFfig7, type = "equal")

postOddsFig7 <- priorOddsFig7 * BFfig7

postProbFig7 <- as.BFprobability(postOddsFig7)

# View input matrices and output calculations:

# Stats for data in Figures 3-7

Data

data3

BFfig3

postProbFig3

data4NvV

BFfig4NvV

postProbFig4NvV

data4NvP

BFfig4NvP

postProbFig4NvP

data4VvP

BFfig4VvP

postProbFig4VvP

data5

BFfig5

postProbFig5

data6NvV

BFfig6NvV

postProbFig6NvV

data6NvP

BFfig6NvP

postProbFig6NvP

data6VvP

BFfig6VvP

postProbFig6VvP

data7

BFfig7

postProbFig7

# Read in historical data with Pan99 in the airborne model [3,4]

# and compare against all possible results obtained from

# 4 donor-recipient pairs in a single experimental replicate.

# Format historical data vs. results as 2x2 matrices (contingency tables).

dataHistVs0of4 <- matrix(data = c(29,3,0,4), nrow = 2, ncol = 2, byrow = TRUE,

dimnames = list(c("HistoricalAirborne","0of4"),

c("Transmission","No transmission")))

dataHistVs1of4 <- matrix(data = c(29,3,1,3), nrow = 2, ncol = 2, byrow = TRUE,

dimnames = list(c("HistoricalAirborne","1of4"),

c("Transmission","No transmission")))

dataHistVs2of4 <- matrix(data = c(29,3,2,2), nrow = 2, ncol = 2, byrow = TRUE,

dimnames = list(c("HistoricalAirborne","2of4"),

c("Transmission","No transmission")))

dataHistVs3of4 <- matrix(data = c(29,3,3,1), nrow = 2, ncol = 2, byrow = TRUE,

dimnames = list(c("HistoricalAirborne","3of4"),

c("Transmission","No transmission")))

dataHistVs4of4 <- matrix(data = c(29,3,4,0), nrow = 2, ncol = 2, byrow = TRUE,

dimnames = list(c("HistoricalAirborne","4of4"),

c("Transmission","No transmission")))

# Calculate Bayes factors for all 2x2 contingency tables [1].

BFv0 <- contingencyTableBF(dataHistVs0of4,sampleType = "indepMulti", fixedMargin = "rows")

BFv1 <- contingencyTableBF(dataHistVs1of4,sampleType = "indepMulti", fixedMargin = "rows")

BFv2 <- contingencyTableBF(dataHistVs2of4,sampleType = "indepMulti", fixedMargin = "rows")

BFv3 <- contingencyTableBF(dataHistVs3of4,sampleType = "indepMulti", fixedMargin = "rows")

BFv4 <- contingencyTableBF(dataHistVs4of4,sampleType = "indepMulti", fixedMargin = "rows")

# Convert Bayes factor to a % likelihood [2].

priorOddsHv0 <- newPriorOdds(BFv0, type = "equal")

postOddsHv0 <- priorOddsHv0 * BFv0

postProbHv0 <- as.BFprobability(postOddsHv0)

priorOddsHv1 <- newPriorOdds(BFv1, type = "equal")

postOddsHv1 <- priorOddsHv1 * BFv1

postProbHv1 <- as.BFprobability(postOddsHv1)

priorOddsHv2 <- newPriorOdds(BFv2, type = "equal")

postOddsHv2 <- priorOddsHv2 * BFv2

postProbHv2 <- as.BFprobability(postOddsHv2)

priorOddsHv3 <- newPriorOdds(BFv3, type = "equal")

postOddsHv3 <- priorOddsHv3 * BFv3

postProbHv3 <- as.BFprobability(postOddsHv3)

priorOddsHv4 <- newPriorOdds(BFv4, type = "equal")

postOddsHv4 <- priorOddsHv4 * BFv4

postProbHv4 <- as.BFprobability(postOddsHv4)

# View input matrices and output calculations:

# Historical data with Pan99 in the airborne model vs. possible

# results of a 4 donor-recipient pair transmission experiment

dataHistVs0of4

BFv0

postProbHv0

dataHistVs1of4

BFv1

postProbHv1

dataHistVs2of4

BFv2

postProbHv2

dataHistVs3of4

BFv3

postProbHv3

dataHistVs4of4

BFv4

postProbHv4

# Read in historical data with Pan99 in the airborne model [3,4]

# and compare against the results shown in Figure 3A

# (transmission to 1 of 8 recipients).

# Format historical data vs. results as 2x2 matrix (contingency table).

dataHistVs3A <- matrix(data = c(29,3,1,7), nrow = 2, ncol = 2, byrow = TRUE,

dimnames = list(c("HistoricalAirborne","Fig 3A Nasal Prep"),

c("Transmission","No transmission")))

# Calculate Bayes factor for the 2x2 contingency table [1].

BFv3A <- contingencyTableBF(dataHistVs3A,sampleType = "indepMulti", fixedMargin = "rows")

# Convert Bayes factor to a % likelihood [2].

priorOddsHv3A <- newPriorOdds(BFv3A, type = "equal")

postOddsHv3A <- priorOddsHv3A * BFv3A

postProbHv3A <- as.BFprobability(postOddsHv3A)

# View input matrices and output calculations:

# Historical data with Pan99 in the airborne model vs.

# results shown in Figure 3A (transmission to 1 of 8 recipients).

dataHistVs3A

BFv3A

postProbHv3A

# References

#

# [1] Jamil T, Ly A, Morey RD, Love J, Marsman M, Wagenmakers E-J.

# Default “Gunel and Dickey” Bayes factors for contingency tables.

# Behavior Research Methods 2017;49:638-652.

# [2] Morey RD. Odds and probabilities using BayesFactor.

# Available at https://cran.r-project.org/web/packages/BayesFactor/

# vignettes/odds_probs.html.Accessed 22 June 2022.

# [3] Bouvier NM, Lowen AC. Animal Models for Influenza Virus Pathogenesis

# and Transmission. Viruses 2010;2:1530-1563.

# [4] Lowen AC, Bouvier NM, Steel J. Transmission in the Guinea Pig Model.

# In: Compans RW, Oldstone MBA, eds. Influenza Pathogenesis and Control

# Volume I. Current Topics in Microbiology and Immunology.

# Cham, Switzerland: Springer International Publishing; 2014:157-183.

- **R Studio console output:**

> # View input matrices and output calculations:

> # Stats for data in Figures 3-7

>

> Data

X X.1 Figure3 Figure4 Figure5 Figure6 Figure7

1 Transmission Nasal Prep 1 0 0 1 0

2 Vehicle NA 1 0 1 NA

3 PBS 2 3 NA 6 4

4 No transmission Nasal Prep 7 7 4 6 4

5 Vehicle NA 7 4 5 NA

6 PBS 2 1 NA 0 0

> data3

Transmission No transmission

Nasal Prep 1 7

PBS 2 2

> BFfig3

Bayes factor analysis

--------------

[1] Non-indep. (a=1) : 1.324074 ±0%

Against denominator:

Null, independence, a = 1

---

Bayes factor type: BFcontingencyTable, independent multinomial

> postProbFig3

Posterior probabilities

--------------

[1] Non-indep. (a=1) : 0.5697211 ±NA%

[2] Indep. (a=1) : 0.4302789 ±NA%

Normalized probability: 1

---

Model type: BFcontingencyTable, independent multinomial

> data4NvV

Transmission No transmission

Nasal Prep 0 7

Vehicle 1 7

> BFfig4NvV

Bayes factor analysis

--------------

[1] Non-indep. (a=1) : 0.4166667 ±0%

Against denominator:

Null, independence, a = 1

---

Bayes factor type: BFcontingencyTable, independent multinomial

> postProbFig4NvV

Posterior probabilities

--------------

[1] Non-indep. (a=1) : 0.2941176 ±NA%

[2] Indep. (a=1) : 0.7058824 ±NA%

Normalized probability: 1

---

Model type: BFcontingencyTable, independent multinomial

> data4NvP

Transmission No transmission

Nasal Prep 0 7

PBS 3 1

> BFfig4NvP

Bayes factor analysis

--------------

[1] Non-indep. (a=1) : 12.375 ±0%

Against denominator:

Null, independence, a = 1

---

Bayes factor type: BFcontingencyTable, independent multinomial

> postProbFig4NvP

Posterior probabilities

--------------

[1] Non-indep. (a=1) : 0.9252336 ±NA%

[2] Indep. (a=1) : 0.07476636 ±NA%

Normalized probability: 1

---

Model type: BFcontingencyTable, independent multinomial

> data4VvP

Transmission No transmission

Vehicle 1 7

PBS 3 1

> BFfig4VvP

Bayes factor analysis

--------------

[1] Non-indep. (a=1) : 4.46875 ±0%

Against denominator:

Null, independence, a = 1

---

Bayes factor type: BFcontingencyTable, independent multinomial

> postProbFig4VvP

Posterior probabilities

--------------

[1] Non-indep. (a=1) : 0.8171429 ±NA%

[2] Indep. (a=1) : 0.1828571 ±NA%

Normalized probability: 1

---

Model type: BFcontingencyTable, independent multinomial

> data5

Transmission No transmission

Nasal Prep 0 4

Vehicle 0 4

> BFfig5

Bayes factor analysis

--------------

[1] Non-indep. (a=1) : 0.36 ±0%

Against denominator:

Null, independence, a = 1

---

Bayes factor type: BFcontingencyTable, independent multinomial

> postProbFig5

Posterior probabilities

--------------

[1] Non-indep. (a=1) : 0.2647059 ±NA%

[2] Indep. (a=1) : 0.7352941 ±NA%

Normalized probability: 1

---

Model type: BFcontingencyTable, independent multinomial

> data6NvV

Transmission No transmission

Nasal Prep 1 6

Vehicle 1 5

> BFfig6NvV

Bayes factor analysis

--------------

[1] Non-indep. (a=1) : 0.4642857 ±0%

Against denominator:

Null, independence, a = 1

---

Bayes factor type: BFcontingencyTable, independent multinomial

> postProbFig6NvV

Posterior probabilities

--------------

[1] Non-indep. (a=1) : 0.3170732 ±NA%

[2] Indep. (a=1) : 0.6829268 ±NA%

Normalized probability: 1

---

Model type: BFcontingencyTable, independent multinomial

> data6NvP

Transmission No transmission

Nasal Prep 1 6

PBS 6 0

> BFfig6NvP

Bayes factor analysis

--------------

[1] Non-indep. (a=1) : 61.28571 ±0%

Against denominator:

Null, independence, a = 1

---

Bayes factor type: BFcontingencyTable, independent multinomial

> postProbFig6NvP

Posterior probabilities

--------------

[1] Non-indep. (a=1) : 0.983945 ±NA%

[2] Indep. (a=1) : 0.01605505 ±NA%

Normalized probability: 1

---

Model type: BFcontingencyTable, independent multinomial

> data6VvP

Transmission No transmission

Vehicle 1 5

PBS 6 0

> BFfig6VvP

Bayes factor analysis

--------------

[1] Non-indep. (a=1) : 35.02041 ±0%

Against denominator:

Null, independence, a = 1

---

Bayes factor type: BFcontingencyTable, independent multinomial

> postProbFig6VvP

Posterior probabilities

--------------

[1] Non-indep. (a=1) : 0.972238 ±NA%

[2] Indep. (a=1) : 0.02776204 ±NA%

Normalized probability: 1

---

Model type: BFcontingencyTable, independent multinomial

> data7

Transmission No transmission

Nasal Prep 0 4

PBS 4 0

> BFfig7

Bayes factor analysis

--------------

[1] Non-indep. (a=1) : 25.2 ±0%

Against denominator:

Null, independence, a = 1

---

Bayes factor type: BFcontingencyTable, independent multinomial

> postProbFig7

Posterior probabilities

--------------

[1] Non-indep. (a=1) : 0.9618321 ±NA%

[2] Indep. (a=1) : 0.03816794 ±NA%

Normalized probability: 1

---

Model type: BFcontingencyTable, independent multinomial

> # View input matrices and output calculations:

> # Historical data with Pan99 in the airborne model vs. possible

> # results of a 4 donor-recipient .... [TRUNCATED]

Transmission No transmission

HistoricalAirborne 29 3

0of4 0 4

> BFv0

Bayes factor analysis

--------------

[1] Non-indep. (a=1) : 377.4 ±0%

Against denominator:

Null, independence, a = 1

---

Bayes factor type: BFcontingencyTable, independent multinomial

> postProbHv0

Posterior probabilities

--------------

[1] Non-indep. (a=1) : 0.9973573 ±NA%

[2] Indep. (a=1) : 0.002642706 ±NA%

Normalized probability: 1

---

Model type: BFcontingencyTable, independent multinomial

> dataHistVs1of4

Transmission No transmission

HistoricalAirborne 29 3

1of4 1 3

> BFv1

Bayes factor analysis

--------------

[1] Non-indep. (a=1) : 22.015 ±0%

Against denominator:

Null, independence, a = 1

---

Bayes factor type: BFcontingencyTable, independent multinomial

> postProbHv1

Posterior probabilities

--------------

[1] Non-indep. (a=1) : 0.9565501 ±NA%

[2] Indep. (a=1) : 0.04344992 ±NA%

Normalized probability: 1

---

Model type: BFcontingencyTable, independent multinomial

> dataHistVs2of4

Transmission No transmission

HistoricalAirborne 29 3

2of4 2 2

> BFv2

Bayes factor analysis

--------------

[1] Non-indep. (a=1) : 2.840645 ±0%

Against denominator:

Null, independence, a = 1

---

Bayes factor type: BFcontingencyTable, independent multinomial

> postProbHv2

Posterior probabilities

--------------

[1] Non-indep. (a=1) : 0.7396271 ±NA%

[2] Indep. (a=1) : 0.2603729 ±NA%

Normalized probability: 1

---

Model type: BFcontingencyTable, independent multinomial

> dataHistVs3of4

Transmission No transmission

HistoricalAirborne 29 3

3of4 3 1

> BFv3

Bayes factor analysis

--------------

[1] Non-indep. (a=1) : 0.6657762 ±0%

Against denominator:

Null, independence, a = 1

---

Bayes factor type: BFcontingencyTable, independent multinomial

> postProbHv3

Posterior probabilities

--------------

[1] Non-indep. (a=1) : 0.3996793 ±NA%

[2] Indep. (a=1) : 0.6003207 ±NA%

Normalized probability: 1

---

Model type: BFcontingencyTable, independent multinomial

> dataHistVs4of4

Transmission No transmission

HistoricalAirborne 29 3

4of4 4 0

> BFv4

Bayes factor analysis

--------------

[1] Non-indep. (a=1) : 0.3228006 ±0%

Against denominator:

Null, independence, a = 1

---

Bayes factor type: BFcontingencyTable, independent multinomial

> postProbHv4

Posterior probabilities

--------------

[1] Non-indep. (a=1) : 0.2440282 ±NA%

[2] Indep. (a=1) : 0.7559718 ±NA%

Normalized probability: 1

---

Model type: BFcontingencyTable, independent multinomial

> # View input matrices and output calculations:

> # Historical data with Pan99 in the airborne model vs.

> # results shown in Figure 3A (transmission .... [TRUNCATED]

Transmission No transmission

HistoricalAirborne 29 3

Fig 3A Nasal Prep 1 7

> BFv3A

Bayes factor analysis

--------------

[1] Non-indep. (a=1) : 2949.02 ±0%

Against denominator:

Null, independence, a = 1

---

Bayes factor type: BFcontingencyTable, independent multinomial

> postProbHv3A

Posterior probabilities

--------------

[1] Non-indep. (a=1) : 0.999661 ±NA%

[2] Indep. (a=1) : 0.0003389808 ±NA%

Normalized probability: 1

---

Model type: BFcontingencyTable, independent multinomial

> # References

> #

> # [1] Jamil T, Ly A, Morey RD, Love J, Marsman M, Wagenmakers E-J.

> # Default “Gunel and Dickey” Bayes factors for continge .... [TRUNCATED]

**References for Supplementary Text 1**

Bouvier NM, Lowen AC. Animal Models for Influenza Virus Pathogenesis and Transmission. Viruses 2010;2:1530-1563.

Jamil T, Ly A, Morey RD, Love J, Marsman M, Wagenmakers E-J. Default “Gunel and Dickey” Bayes factors for contingency tables. Behavior Research Methods 2017;49:638-652.

Lowen AC, Bouvier NM, Steel J. Transmission in the Guinea Pig Model. In: Compans RW, Oldstone MBA, eds. Influenza Pathogenesis and Control - Volume I. Current Topics in Microbiology and Immunology. Cham, Switzerland: Springer International Publishing; 2014:157-183.

Morey RD. Odds and probabilities using BayesFactor. Available at https://cran.r-project.org/web/packages/BayesFactor/vignettes/odds_probs.html. Accessed 22 June 2022.
